# Moving Object Localization Using Optical Flow for Pedestrian Detection from a Moving Vehicle

**DOI:** 10.1155/2014/196415

**Published:** 2014-07-10

**Authors:** Joko Hariyono, Van-Dung Hoang, Kang-Hyun Jo

**Affiliations:** Graduate School of Electrical Engineering, University of Ulsan, Ulsan 680-749, Republic of Korea

## Abstract

This paper presents a pedestrian detection method from a moving vehicle using optical flows and histogram of oriented gradients (HOG). A moving object is extracted from the relative motion by segmenting the region representing the same optical flows after compensating the egomotion of the camera. To obtain the optical flow, two consecutive images are divided into grid cells 14 × 14 pixels; then each cell is tracked in the current frame to find corresponding cell in the next frame. Using at least three corresponding cells, affine transformation is performed according to each corresponding cell in the consecutive images, so that conformed optical flows are extracted. The regions of moving object are detected as transformed objects, which are different from the previously registered background. Morphological process is applied to get the candidate human regions. In order to recognize the object, the HOG features are extracted on the candidate region and classified using linear support vector machine (SVM). The HOG feature vectors are used as input of linear SVM to classify the given input into pedestrian/nonpedestrian. The proposed method was tested in a moving vehicle and also confirmed through experiments using pedestrian dataset. It shows a significant improvement compared with original HOG using ETHZ pedestrian dataset.

## 1. Introduction

Vision-based environment detection methods have been actively developed in robot vision. Detecting pedestrian is one of the essential tasks for understanding environment. Pedestrian detection in images could be used in video surveillance systems and driver assistance systems. It is more challenging to detect moving objects or pedestrian in order to avoid an obstacle and control locomotion of the vehicle in the real-world environment.

In the past few years, moving object and pedestrian detection methods for a mobile robot or moving vehicle have been actively developed. For a practical real-time pedestrian detection system, Gavrila and Munder [1] employed hierarchical shape matching to find pedestrian candidates from moving vehicle. Their method uses a multicue vision system for the real-time detection and tracking of pedestrians. Nishida and Kurita [2] applied SVM with the automated selection process of the components by using AdaBoost. These researches show that the selection of the components and their combination are important to get a good pedestrian detector.

Many local descriptors are proposed for object recognition and image retrieval. Mikolajczyk and Schmid [3] compared the performance of several local descriptors and showed that the best matching results were obtained by the scale invariant feature transform (SIFT) descriptor [4]. Dalal et al. [5, 6] proposed a human detection algorithm using histograms of oriented gradients (HOG) which are similar to the features used in the SIFT descriptor. HOG features are calculated by taking orientation histograms of edge intensity in a local region. They are designed by imitating the visual information processing in the brain and have robustness for local changes of appearances and position. Dalal et al. extracted the HOG features from all locations of a dense grid on an image region and the combined features are classified by using linear SVM. They showed that the grids of HOG descriptors significantly out-performed existing feature sets for human detection. Kobayasi et al. [7] proposed selected feature of HOG using PCA to decrease the number of features. It could reduce the number of features by less than half without lowering the performance.

Moving object detection and motion estimation methods using the optical flow for a mobile robot also have been actively developed. Talukder et al. [8] proposed a qualitative obstacle detection method that was proposed using the directional divergence of the motion field. The optical flow pattern was investigated in perspective camera and this pattern was used for moving object detection. Also, real-time moving object detection method was presented during translational robot motion.

Several researchers also developed methods for egomotion estimation and navigation from a mobile robot using an omnidirectional camera [9, 10]. They tried to measure camera egomotion itself using omnidirectional vision. They used Lucas Kanade optical flow tracker and obtained corresponding features of background in the consecutive two omnidirectional images. The motion of feature points analysis is used to calculate camera egomotion, however they didn't use for moving object detection. They set up an omnidirectional camera on a mobile robot and obtained panoramic image transformed from omnidirectional image. They obtained camera egomotion compensated frame difference based on an affine transformation of two consecutive frames where corner features were tracked by Kanade-Lucas-Tomasi (KLT) optical flow tracker [11]. However, detecting moving objects resulted in a problem that only one affine transformation model could not represent the whole background changes since the panoramic image has many local changes of scaling, translation, and rotation of pixel groups. For this problem, our previous work [12] proposed that each affine transformation of local pixel groups should be tracked by KLT tracker. The local pixel groups are not a type of image features such as corner or edge. We use grid windows-based KLT tracker by tracking each local sector of panoramic image (Figure 2) while other methods use sparse features-based KLT tracker. Therefore, we can segment moving objects in panoramic image by overcoming the nonlinear background transformation of panoramic image [13].

## 2. Related Works

Proposed method is inspired by the works on pedestrian detection from moving vehicle [1, 8], using optical flow [11] and egomotion estimation [9], we called it is egomotion compensate [12]. Pedestrian as a moving object is extracted from the relative motion by segmenting the region representing the same optical flows after compensating the egomotion of the camera. To obtain the optical flow, image is divided into grid windows and affine transformation is performed according to each window, so that conformed optical flows are extracted. The regions of moving object are detected as transformed objects are different from the previously registered background. Morphological process is applied to get the candidate region of human shape. In order to recognize the object, HOG features were extracted on a candidate region and classified using linear SVM [5, 14]. The HOG feature vectors are used as an input of linear SVM to classify the given input into pedestrian/nonpedestrian. For the performance evaluation, comparative study was presented in this paper.

## 3. Moving Object Segmentation

**Figure 1:**
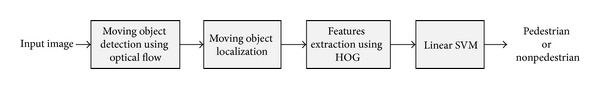
The overview of the pedestrian detection algorithm.

**Figure 2:**
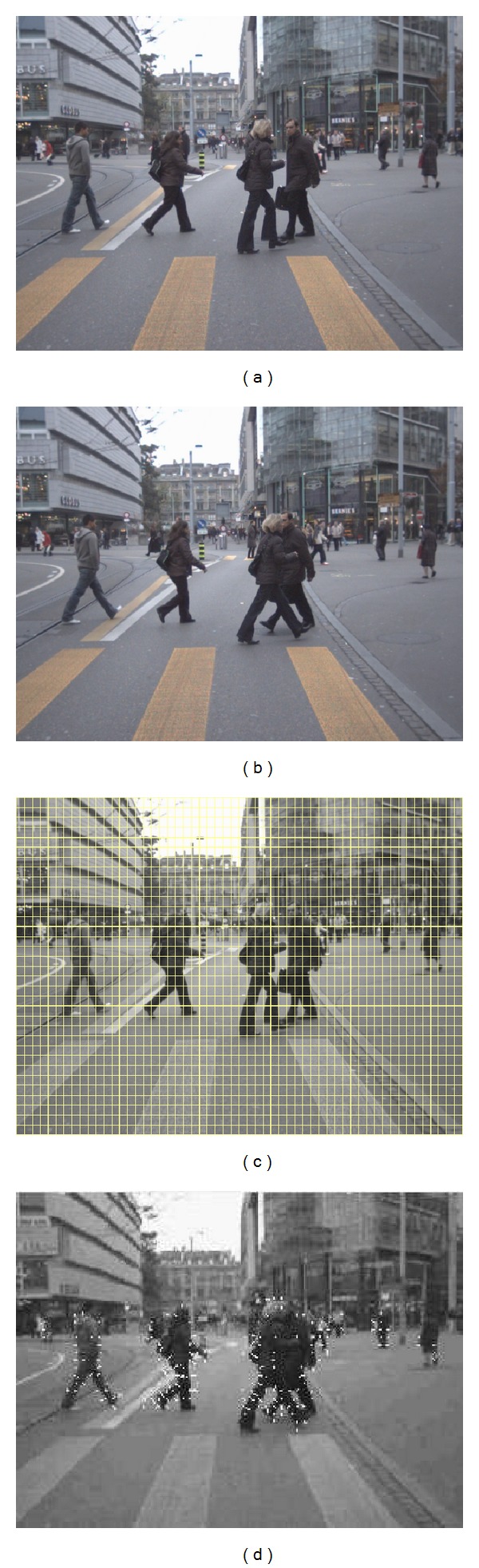
From two consecutive image sequences (a) and (b), we decide grid windows (c) and track each window in the next consecutive image (d).

This section presents how to detect moving object from the camera mounted on the vehicle. In order to obtain moving object area from video or sequent of images, it is not easy to segment out only moving object area, because the camera moving is also caused by camera egomotion. So, we proposed a method to deal with this situation [12]. We used optical flow analysis to segment independent motion of moving object from egomotion caused by camera. It is called egomotion compensated. The optical flow caused by independent motion of moving object will have different pattern compared with flow caused by egomotion from camera; then, we localize those different pattern as a region of moving object. This region is candidate of detected human/pedestrian after we apply HOG. The overview of the pedestrian detection algorithm is shown in Figure 1.

### 3.1. Egomotion Compensated

In our previous work [12], we apply KLT optical flow tracker [11] in order to deal with several conditions. Brightness constancy, which is projection of the same point, looks the same in every frame; small motion that points do not move very far and spatial coherence that points move like their neighbors.

The frame difference represents all motions caused by camera egomotion and moving object in the scene. It needs to compensate this effect from frame difference to segment out only independent motion of moving object, so how much the background image has been transformed in two sequences of images. Affine transformation represents the pixel movement between two sequence images as follows:

(1) $$\begin{array}{c} P^{\prime }=AP+t, \end{array}$$

where *P* and *P*′ are pixel location in the first and the second frame. *A* is transformation matrix and *t* is translation vector. Affine parameters are calculated by least square method using at least three corresponding features in two images.

**Figure 3:**
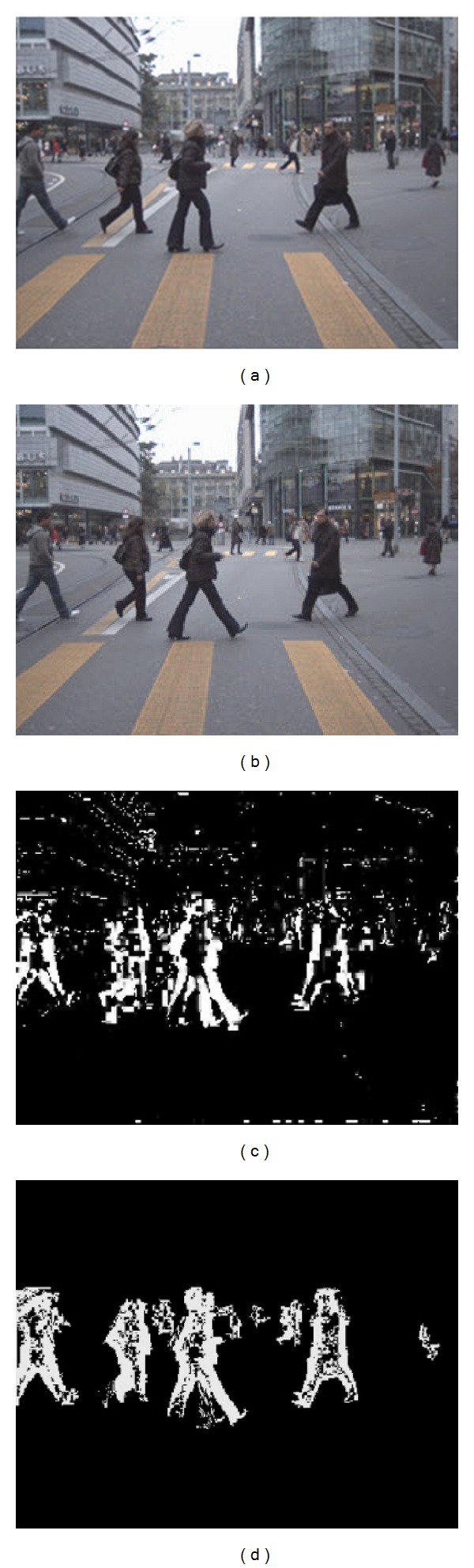
From two consecutive images (a) and (b), we applied frame difference (c) and comparing when we applied frame difference with egomotion compensated (d).

In this work, the original input images are converted to grayscale images, and one channel intensity pixel value from the input images is obtained. Then, use two consecutive images which are divided into grid cells of size 14 × 14 pixels; then compare and track each cell in current frame to find corresponding cell in the next frame. The cell that has the most similar intensity value in a group will be selected as corresponding value. Using method from [11], find the motion distance of each pixel in a group of cells, the motion *d* in *x*-axis and *y*-axis of each cell *g*_*t*−1_(*i*, *j*), by finding most similar cell *g*_*t*_(*i*, *j*) in the next frame,

(2) $$\begin{array}{c} g_{t-1}\left(i,j\right)=g_{t}\left(i+d_{x},j+d_{y}\right), \end{array}$$

where *d*_*x*_ and *d*_*y*_ are motion distances in *x*-axis and *y*-axis, respectively. At least three corresponding features are used to estimate the affine parameters using the least square method. Equation (2) is rewritten by affine transformation of each pixel in the same cell as follows:

(3) $$\begin{array}{c} I_{t}\left(x,y\right)=AI_{t-1}\left(x,y\right)+d, \end{array}$$

where *I*_*t*_(*x*, *y*) and *I*_*t*−1_(*x*, *y*) are vector 2 × 1 which represent pixel location in the current and previous frame, respectively; *A* is 2 × 2 projection matrix and *d* is 2 × 1 translation vector. The results are shown in Figure 3.

To obtain the camera egomotion compensated, frame difference is applied in two consecutive input images by calculating based on the tracked corresponding pixel cells using

(4) $$\begin{array}{c} I_{d}\left(x,y\right)=\left|I_{t-1}\left(x,y\right)-I_{t}\left(x,y\right)\right|, \end{array}$$

where *I*_*d*_(*x*, *y*) is a pixel cell located at (*x*, *y*) in the grid cell.

Suppose that two consecutive images shown in Figures 3(a) and 3(b) cannot segment out moving object using frame difference Figure 3(c), however when we apply frame difference with egomotion compensate could obtain moving objects area shown in Figure 3(d).

### 3.2. Moving Object Localization

Each pixel output from frame difference using egomotion compensated cannot show clearly as silhouette. It just gives information of motion areas from moving objects. Those moving areas are applied to morphological process to obtain region of moving object and noise removal.

Ideally, we would seek to devise a region segmentation algorithm that accurately locates the bounding boxes of the motion regions in the difference image. Given the sparseness of the data, however, accurate segmentation would involve the enforcement of multiple constraints, making fast implementation difficult. To achieve faster segmentation, we assumed the fact that humans usually appear in upright positions and conclude that segmenting the scene into vertical strips is sufficient most of the time. In this work, we define detected moving objects that are represented by the position in width in *x*-axis. Using projection histogram *h*_*x*_ by pixel voting vertically projects image intensities into *x*-coordinate.

Adopting the region segmentation technique proposed in [15], we define the region using boundary saliency. It measures the horizontal difference of data density in the local neighborhood. The local maxima, which correspond to where maximal change in data density occurs, are candidates for region boundaries of pedestrian in moving object detection.

## 4. Feature Extraction

In this section, we present how we extract feature from candidate region obtained from previous section. In this work, we use histogram of oriented gradients (HOG) to extract features from moving object area localization. Local object appearance and shape usually can be characterized well by the distribution of local intensity gradients or edge direction. HOG features are calculated by taking orientation histograms of edge intensity in local region.

### 4.1. HOG Features

**Figure 4:**
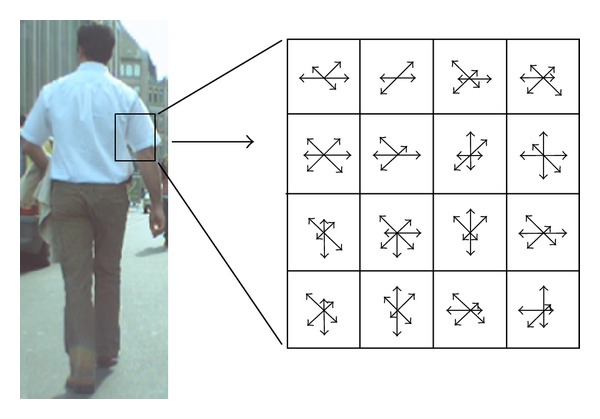
Extraction process of HOG features. The HOG features are extracted from local regions with 16 × 16 pixels. Histograms of edge gradients with 8 orientations are calculated from each of the 4 × 4 local cells.

In this work, we extract HOG features from 16 × 16 local regions as shown in Figure 4. At first, we use Sobel filter to obtain the edge gradients, and orientations were calculated from each pixel in this local region. The gradient magnitude *m*(*x*, *y*) and orientation *θ*(*x*, *y*) are calculated using directional gradients *dx*(*x*, *y*) and *dy*(*x*, *y*) computed by Sobel filter as

(5) $$\begin{array}{c} m\left(x,y\right)=\sqrt{dx{\left(x,y\right)}^{2}-dy{\left(x,y\right)}^{2}}, \end{array}$$

(6) θ(x,y)= {tan−1⁡(dy(x,y)dx(x,y))−π, if  dx(x,y)<0,dy(x,y)<0,tan−1⁡(dy(x,y)dx(x,y))+π, if  dx(x,y)<0,dy(x,y)>0,tan−1⁡(dy(x,y)dx(x,y)), otherwise.

The local region is divided into small spatial or cell, each size is 4 × 4 pixels. Histograms of edge gradients with 8 orientations are calculated from each of the local cells. Then the total number of HOG features becomes 128 = 8 ×(4 × 4) and they constitute a HOG feature vector. To avoid sudden changes in the descriptor with small changes in the position of the window and to give less emphasis to gradients that are far from the center of the descriptor, a Gaussian weighting function with* σ *equal to one-half of the width of the descriptor window is used to assign a weight to the magnitude of each pixel.

A vector of HOG feature represents local shape of an object, it has edge information at plural cells. In flatter regions like a ground or a wall of a building, the histogram of the oriented gradients has flatter distribution. On the other hand, in the border between an object and background, one of the elements in the histogram has a large value and it indicates the direction of the edge. Even though the images are normalized to position and scale, the positions of important features will not be registered with the same grid positions. It is known that HOG features are robust to the local geometric and photometric transformations. If the translations or rotations of the object are much smaller than the local spatial bin size, their effect is small.

**Figure 5:**
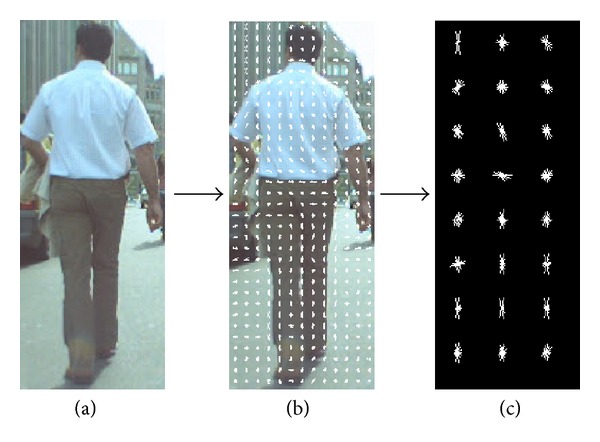
From a candidate input image of size 150 × 382 (a), HOG features are extracted from all locations on the candidate region of an input image with 16 × 16 pixels region (b), and the result is shown in (c).

Dalal and Triggs [5] extracted a set of HOG feature vectors from all locations in an image grid and that are used for classification. In this work, we just extract the HOG features from all locations on the candidate region localization from an input image as shown in Figure 5.

### 4.2. Linear SVM Classifier

In the human detection algorithm proposed by Dalal and Triggs [5], the HOG features are extracted from all locations of a dense grid and the combined features are classified using linear support vector machine (SVM). HOG shows significantly outperformed existing feature sets for human detection. This work also used the linear SVM to perform work in various data classification tasks. Let {*f*_*i*_,*t*_*i*_}_*i*=1_^*N*^(*f*_*i*_ ∈ *R* ^*D*^, *t*_*i*_ ∈ {−1, 1}) be the given training sample in D-dimensional feature space. The classification function is given as

(7) $$\begin{array}{c} z=\mathrm{sign}\left(\omega^{T}f_{i}-h\right), \end{array}$$

where *ω* and *h* are the parameters of the model. For the case of soft-margin SVM, the optimal parameters are obtained by minimizing

(8) $$\begin{array}{c} L\left(\omega ,\xi \right)=\frac{1}{2}{||\omega ||}^{2}+C\overset{N}{\underset{i=1}{\sum }}\xi_{i} \end{array}$$

under the constraints

(9) $$\begin{array}{c} \xi_{i}\geq 0,\;\;t_{i}\left(\omega^{T}f_{i}-h\right)\geq 1-\xi_{i}\;\left(i=1,\ldots ,N\right), \end{array}$$

where  *ξ*_*i*_  (≥ 0) is the error of the *i*th sample measured from the separating hyperplane and *C* is the hyperparameter which controls the weight between the errors and the margin. The dual problem of (8) is obtained by introducing Lagrange multipliers **α** = (*α*_1_,…, *α*_*N*_), *α*_*k*_ ≥ 0 as

(10) $$\begin{array}{c} L_{D}\left(\alpha \right)=\overset{N}{\underset{i=1}{\sum }}\alpha_{i}-\frac{1}{2}\overset{N}{\underset{i,j=1}{\sum }}\alpha_{i}\alpha_{j}t_{i}t_{j}f_{i}^{T}f_{i} \end{array}$$

under the constraints

(11) $$\begin{array}{c} \overset{N}{\underset{i=1}{\sum }}\alpha_{i}t_{i}=0,\;0\leq \alpha_{i}\;\left(i=1,\ldots ,N\right). \end{array}$$

By solving (10), the optimum function is obtained as

(12) $$\begin{array}{c} z=\mathrm{sign}\left(\underset{i\in S}{\sum }\alpha_{i}^{*}t_{i}f_{i}^{T}f_{i}-h^{*}\right), \end{array}$$

where *S* is the set of support vectors.

To get a good classifier, we have to search the best hyperparameter *C*. The cross-validation is used to measure the goodness of the linear SVM classifier.

## 5. Experimental Results

In this work, our vehicle system is run in outdoor environment with speed that varies from around 0 to 50 kilometers per hour and detected object moving surround its path. Proposed algorithm was programmed in MATLAB and executed on a Pentium 3.40 GHz, 32-bit operating system with 8 GB random access memory. The proposed algorithm was evaluated by using five sequences of images from ETHZ pedestrian datasets which contain around 5,000 images of pedestrians in city scenes [15]. It contains only front or back views with relatively limited range of poses and the position and the height of human in the image are almost adjusted. The size of the image is 640 × 480 pixels. For the training process, we used person INRIA datasets [5]. These images were used for positive samples in the following experiments. The negative samples were originally collected from images of sky, mountain, airplane, building, and so forth. The number of images is 3,000. From these images, 1,000 person images and 2,000 negative samples were used as training samples to determine the parameters of the linear SVM. The remaining 100 pedestrian images and 200 negative samples were used as test samples to evaluate the recognition performance of the constructed classifier.

We studied methods for detecting human, and one of the objectives of this work is that we want a method that can detect people reliably whether they are moving or not. We were concerned that it might be sensitive to the relative proportion of moving and of static people in the videos. We check reliability of the proposed method that the combination of optical flow and HOG not only on the pure video contain of moving object, but also on objects without moving on the sequent images again with static object flows being zero. The results are diluting the fraction of motion regions naturally reduces the advantage of the combination of methods relative to the static ones; however, using the combination of methods, the relative ranking of the methods remains unchanged.  Table 1 shows that when we used on relatively the objects without moving on the images for which there are a less flow field, the best combination of methods detectors do marginally better than the best of original HOG detectors done.

The reliability of our moving object detection system was evaluated whether it still works well in the case if the vehicle ran in varying speed. Outdoor application with speed of vehicle that varies from around 0 to 50 kilometers per hour was performed; then we evaluated the proposed window cells based flow estimation which are still visible at several levels. We tested reliability of the window cells for optical flow tracking in several sizes; it will determines from the relative distance of the object from the camera, so that we consider to choose the flow field window tracking which is more accurate for larger people and also well tracking for smaller people in the image. As a counterweight parameter, computational cost was considered for performance balancing.  Table 2 shows the miss detection rate and computational cost of several windows size. However, 10 × 10 cells are the lowest on the miss detection rate but the slowest in computational cost; size 14 × 14 is selected based on low in miss detection rate and faster computational speed.

After all, we implemented original HOG by Dalal et al. using those datasets; the recognition rate for test dataset is 98.3%. Then, we test the combination of methods based on optical flow and HOG feature. HOG feature vectors were extracted from all locations of the grid for each training sample. Then, the selected feature vectors were used as input of the linear SVM. The selected subsets were evaluated by cross validation. Also, we evaluated the recognition rates of the constructed classifier using test samples.

**Figure 6:**
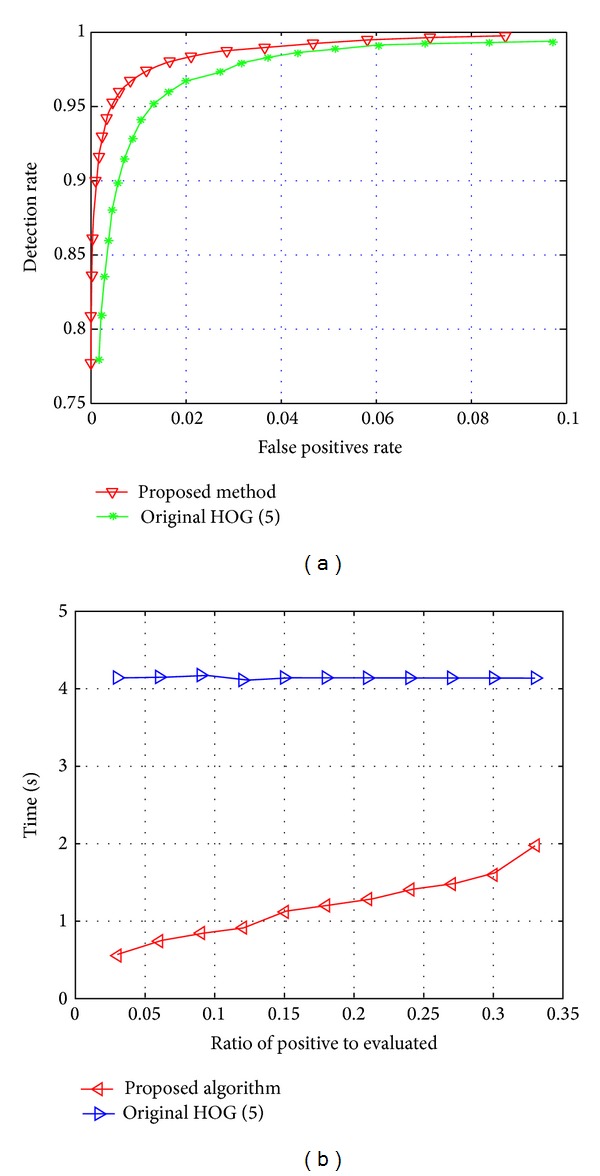
Comparison result when we tested our proposed method and original HOG by Dalal et al. (a) Comparison of detection rate and (b) comparison of time consumption.

**Figure 7:**
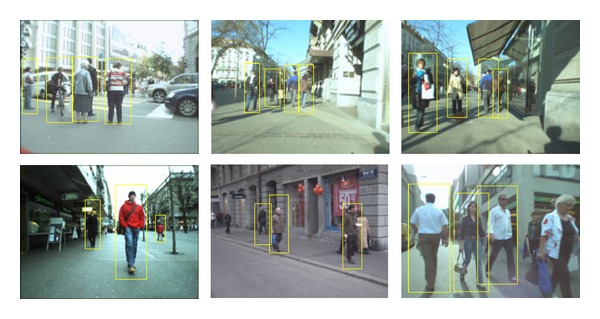
Successful moving objects detection results.

**Figure 8:**
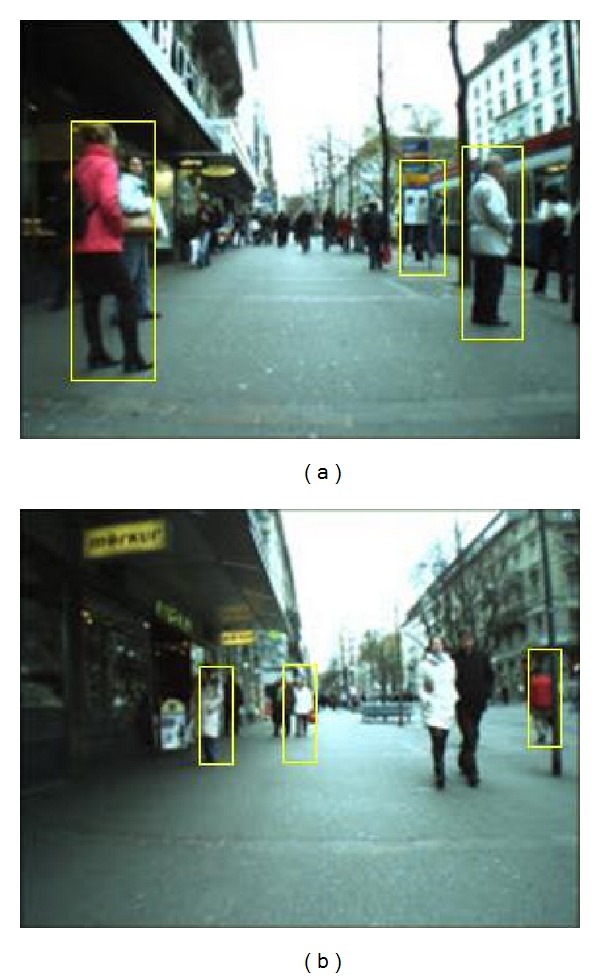
(a) False positives detection and (b) false negative detection.

The relation between the detection rates and the number of false positive rate is shown in Figure 6. The best recognition rate, 99.3%, was obtained at 0.09 false positive rates. It means that we obtain higher detection rate with smaller false positives rate. The computational cost also reduces eight times better when we use small ratio of positive to evaluated data. However, if we increase the number of ratios it also reduces time consuming significantly. The detection results are shown in Figure 7 and false detection is shown in Figure 8.

## 6. Conclusion

This paper addressed the problem for detecting pedestrian from moving vehicle using optical flow and HOG. The moving object is segmented out through the relative evaluation of optical flows to compensate egomotion of camera. Morphological process is applied to get the candidate region of pedestrian. In order to recognize the object, HOG features were extracted on a candidate region and classified using linear SVM. The HOG feature vectors are used as an input of linear SVM to classify the given input into pedestrian/nonpedestrian. The proposed algorithm achieved comparable results compared with original HOG and also reduces computational cost significantly using moving object localization. In the future work, we consider the combination methods [16] compared with modification of HOG, such as LBP HOG and feature selection HOG.

## Figures and Tables

**Table 1:** The detection rates of various detectors.

False positive rate	0.02	0.04	0.06	0.08	0.1
HOG	0.965	0.980	0.985	0.990	0.992
Proposed method	0.980	0.986	0.989	0.992	0.994

**Table 2:** The miss rates of various cell windows size.

Cell window size	False positive rate (0.09)	Computational cost (fps)
10 × 10	0.023	11.89
12 × 12	0.024	11.97
14 × 14	0.024	12.35
16 × 16	0.026	12.55
18 × 18	0.028	12.83
